# 3D-printed lightweight dorsal skin fold chambers from PEEK reduce chamber-related animal distress

**DOI:** 10.1038/s41598-022-13924-5

**Published:** 2022-07-08

**Authors:** Wentao Xie, Matthias Lorenz, Friederike Poosch, Rupert Palme, Dietmar Zechner, Brigitte Vollmar, Eberhard Grambow, Daniel Strüder

**Affiliations:** 1 Institute for Experimental Surgery, Rostock University Medical Center, 18057 Rostock, Germany; 2 Department of Vascular and Thyroid Surgery, Department of General Surgery, The First Affiliated Hospital of Anhui Medical University, Hefei, 230022 China; 3 Faculty of Engineering, Technology, Business and Design, University of Applied Sciences, 23966 Wismar, Germany; 4 Department of Otorhinolaryngology, Head and Neck Surgery “Otto Koerner”, Rostock University Medical Center, 18057 Rostock, Germany; 5 Unit of Physiology, Pathophysiology and Experimental Endocrinology, Department of Biomedical Sciences, University of Veterinary Medicine Vienna, 1210 Vienna, Austria; 6 Department of General, Visceral, Thoracic, Vascular and Transplantation Surgery, Rostock University Medical Center, Schillingallee 35, 18057 Rostock, Germany

**Keywords:** Experimental models of disease, Medical research, Preclinical research

## Abstract

The dorsal skinfold chamber is one of the most important in vivo models for repetitive longitudinal assessment of microcirculation and inflammation. This study aimed to refine this model by introducing a new lightweight chamber made from polyetheretherketone (PEEK). Body weight, burrowing activity, distress, faecal corticosterone metabolites and the tilting angle of the chambers were analysed in mice carrying either a standard titanium chamber or a PEEK chamber. Data was obtained before chamber preparation and over a postoperative period of three weeks. In the early postoperative phase, reduced body weight and increased faecal corticosterone metabolites were found in mice with titanium chambers. Chamber tilting and tilting-related complications were reduced in mice with PEEK chambers. The distress score was significantly increased in both groups after chamber preparation, but only returned to preoperative values in mice with PEEK chambers. In summary, we have shown that light chambers reduce animal distress and may extend the maximum dorsal skinfold chamber observation time. Chambers made of PEEK are particularly suitable for this purpose: They are autoclavable, sufficiently stable to withstand rodent bites, inexpensive, and widely available through 3D printing.

## Introduction

**Figure 1:**
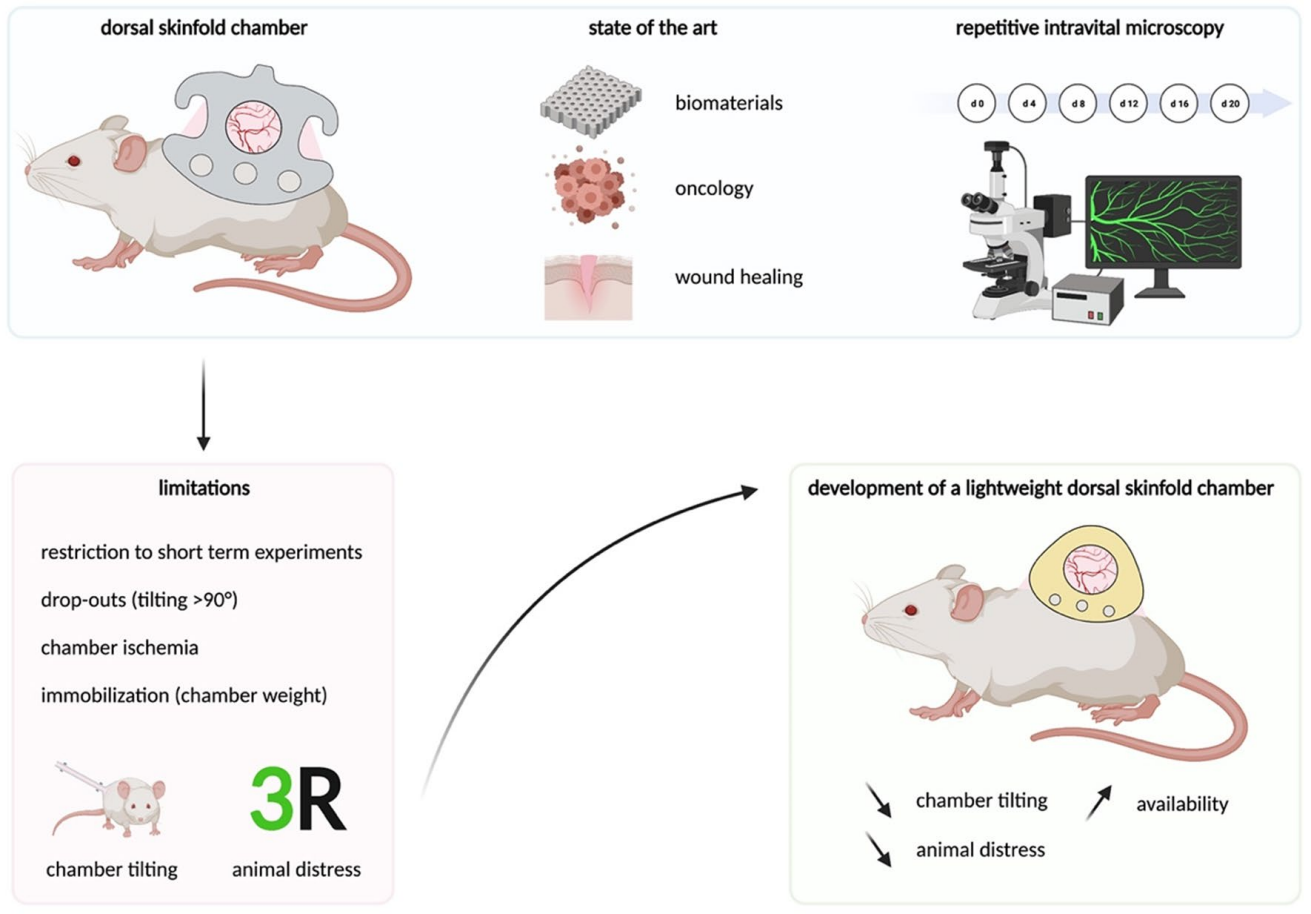
Application and limitations of the dorsal skinfold chamber. The model enables continuous observation of microvascular parameters by intravital microscopy. The major limitations are animal distress and restriction to short-term experiments. To improve animal welfare and extend the observation time, a lightweight, 3D-printable PEEK chamber was developed. Figure created with BioRender.com.

The dorsal skinfold chamber is an essential model for in vivo microcirculation analysis^1^. Researchers investigated inflammation^2–4^, thrombogenesis^5–7^, wound healing^8,9^, angiogenesis, biomaterials^10^ and tumor vascularisation^11–13^ using the dorsal skinfold chamber^14–16^. Repetitive intravital visualisation of the microvascular dynamics is the major advantage of the model (Fig. 1).

Repetitive intravital microscopy without repetitive surgery requires the continuous exposition of the prepared vascular bed. The skinfold chamber on the back of the laboratory animal ensures optimal conditions for repetitive intravital microscopy^17^. The standard chamber comprises two titanium frames fixing the extended dorsal skin in the back's midline. During chamber implantation, the first frame is sutured to one side of the dorsal skinfold. The skin, the subcutaneous tissue, and the striated panniculus carnosus muscle on one side of the dorsal skinfold are removed. Then, microsurgery exposes the vessels of the opposite panniculus carnosus muscle. Finally, screws are punched through the dorsal skinfold to connect the second frame of the chamber and the observation window is filled with saline followed by sealing with a coverslip.

The major limitation of the model is the physical burden of the chamber, which leads to animal immobilization and distress. The titanium dorsal skinfold chamber is 4 cm long, 3 cm high, and weighs 3.8 g. The weight matches up to 20% of the mouse's body weight (20–30 g). Weight and skin stretching may lead to restricted breathing, immobilization, and pain. The severity of any dorsal skinfold chamber experiment is considered at least moderate. Therefore, the reputation of the model as a standard in microvascular research has been contested in the context of 3R (refinement, reduction, replacement)^18^.

Lateral tilting of the dorsal skinfold chamber is another important limitation and strongly related to animal distress^17^. Weight, the chamber's high center-of-gravity, and overstretching of the skin (in particular at the fixation screws) lead to lateral tilting of the chamber in the second week after dorsal skinfold chamber preparation. Tilting comprises perfusion and causes animal distress (immobilization, pain). Chamber tilting of > 50° must be scored in the severity assessment and tilting of > 100° considered as an abort criterion. Therefore, experiments of up to 21 days are associated with high dropouts (20% in the third week) and low reliability (infections, ischemia). Experiments of longer than 21 days are impossible with standard chambers^19,20^.

To overcome these limitations, the chambers were continuously revised. Schreiter et al. reviewed the developments until 2017^1^. While 63% of the dorsal skinfold chamber studies were from German-speaking countries and mostly used titanium chambers, smaller titanium chambers are already standard in the US^1^. Research groups from Sweden and Asia proposed more advanced plastic chambers made from plexiglass, dacron and polyetheretherketone (PEEK)^1^.

For many years, PEEK has been used successfully in medicine as a replacement material for titanium to fabricate surgical devices, implants and prostheses^21,22^. PEEK is a linear, semi-crystalline polymer that exhibits excellent mechanical and thermal properties. PEEK is bioinert and durable (lack of thermal aging and chemical resistance).

Although many publications point out disadvantages of large titanium chambers, they are still used in most studies: Between 2017 and 2021, only four of 76 studies used non-titanium chambers (70 titanium, 3 plastic, 1 steel wire, 2 no information given; Suppl. Table S1)^23–26^.

One factor in the limited distribution of improved dorsal skinfold chamber is that previous research on chamber refinement missed quantifying the actual impact on animal distress. Existing titanium chamber stocks and poor availability of plastic chambers are further factors, which hindered comprehensive implementation of refined dorsal skinfold chambers.

The goal of the present study was the design and evaluation of a lightweight PEEK dorsal skinfold chamber to reduce animal distress and preserve repetitive intravital microscopy quality. We introduce a 3D printed design with unrestricted access and evaluate animal distress compared with traditional chambers. Therefore, the pilot study assessed specific dorsal skinfold chamber parameters (intravital microscopy quality, chamber tilting) and general distress parameters (weight loss, corticosterone levels, behaviour, distress score). As bridging technology, the PEEK chamber may improve future in vivo research until sufficient in vitro and in virtuo models are established.

## Results

This study examined animal distress of standard titanium dorsal skinfold chambers compared to a 3D printed design made of PEEK. Preparation of the chamber and intravital microscopy were comparable. PEEK chambers showed no signs of bites, chews or any other manipulation by the mice. In terms of lateral chamber tilting, animal weight loss and corticoid levels, the PEEK chamber was markedly superior.

**Figure 2:**
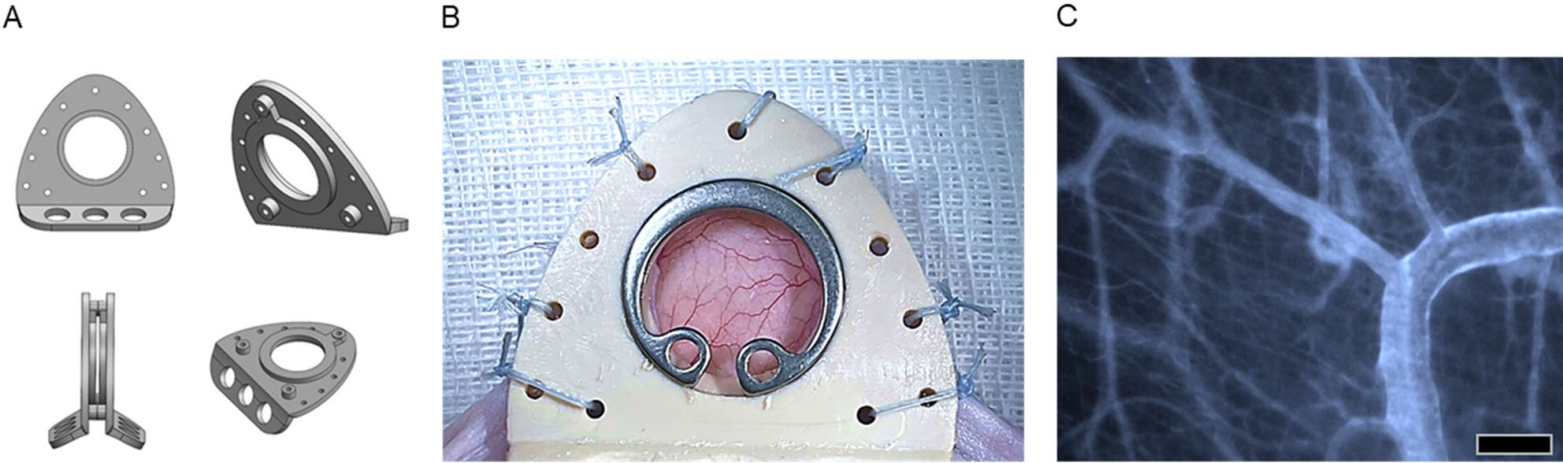
Exemplary design of the PEEK chamber. (**A**) Model of the scaled-down and 3D-printable PEEK chamber (created with SolidWorks). (**B**) The extrusion printed PEEK chamber was ground to remove superficial irregularities and implantated into the test animal. (**C**) Representative intravital microscopy image of a PEEK chamber on day 3. Bar represents 100 µm.

The new PEEK chamber was designed lower and lighter than titanium chambers (approx. 1.5 g instead of 3.8 g). The chamber was 3D-printed cost-effectively according to a reproducible protocol (€5.30 /chamber). The costs were lower compared to milled titanium chambers (€30–€110). The design and the low weight allowed the PEEK chamber to be secured with sutures (Table 1). Robust suture material (FiberWire, Arthrex, Munich, Germany) attached with multiple knots reliably fixed the chamber for 21 days (Fig. 2). The traumatic transdermal insertion of titanium screws was obsolete. The duration and difficulty of chamber implantation was comparable to the titanium chamber (20 min). PEEK is bioinert and autoclavable; wound infections did not occur in the pilot study, even with repeated use of autoclaved chambers.

**Table 1:** Comparison of the titanium and PEEK chamber.

	Titanium chamber	PEEK chamber​​
Weigth (g)	3.8	1.5
Height (mm)	36 × 24	24 × 20
Transdermal screws	3	–
Price (€)	30–110	5

**Figure 3:**
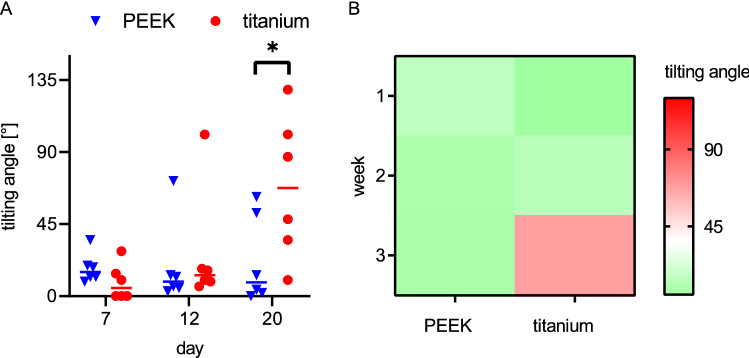
Lateral chamber tilting. PEEK and titanium chamber tilting angles over time are given as individual values (**A**) and as a heatmap indicating high risk for lateral chamber tilting (**B**). PEEK chambers significantly reduced lateral tilting in the third week, while tilting in both chambers was comparable for 12 days. This is further visualized in the heat map (**B**). Differences between the groups were analysed by multiple t-tests (Holm-Sidak), *p < 0.05, PEEK: n = 6, titanium: n = 6.

PEEK chambers significantly reduced lateral tilting in the third week (Fig. 3). In the first week, both preparations were stable in the median plane; the titanium group even showed slightly less tilting (PEEK: 15°/9°–35°; titanium: 5°/0°–28°, p = 0.1688; median/95% confidence interval). None of the chambers tilted by > 45° in the first week. In the second week, one PEEK chamber tilted moderately by 72° and one titanium chamber tilted severely by 102°. The other five of six chambers in the respective groups remained stable with less than 20° deviation. In the third week, the deviation of the moderately tilted PEEK chamber remained stable, and another PEEK chamber tilted likewise. Four of six PEEK chambers showed no deviation even after 21 days. In the titanium group, however, the tilting angle increased markedly in five of six animals. In three of six titanium chamber animals, the chamber deviated to about 90° and the skin around the screws had stretched to large defects. The median tilting of the titanium chambers was significantly greater than tilting of the PEEK chambers in the third week (PEEK: 8.5°/0°–62°; titanium: 67°/10°–129°, p < 0.05).

**Figure 4:**
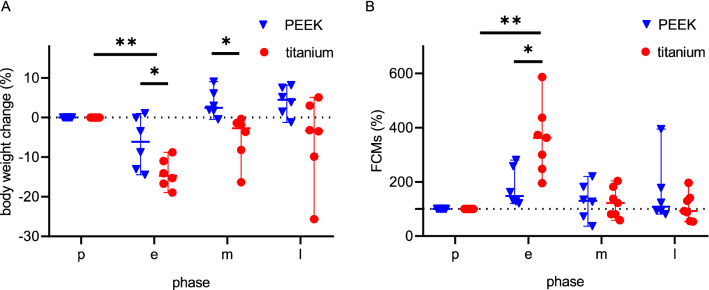
Changes in body weight and faecal corticosterone metabolite (FCM) concentrations after dorsal skinfold chamber implantation. Individual values are given over time in a preoperative phase (p), early postoperative phase (e), middle postoperative phase (m) and late postoperative (l) phase. Body weight change (**A**) between each time point was analysed by RM one-way ANOVA on ranks followed by Dunn’s correction, and the difference between each group was analysed by unpaired t test followed by two-tailed P value tests. FCM concentrations (**B**) between each time point was analysed by Friedman test followed by Dunn’s correction, and the difference between each group was analysed by unpaired t test followed by two-tailed P value tests. *p < 0.05; **p < 0.05; PEEK: n = 6, titanium: n = 6.

Postoperative weight loss was significantly reduced in PEEK chamber mice (Fig. 4A). While significant weight loss occurred postoperatively in all animals, weight loss was lower with PEEK chambers (PEEK: − 6.12%/− 14.46 to 1.06%; titanium: − 14.69%/− 18.95 to 8.77%; p < 0.05). The body weight of PEEK chamber mice recovered already in the middle phase (2.46%/− 0.47 to 9.07%). Titanium chamber animals did not fully recover from the high initial weight loss until the late phase (− 3.33%/− 25.68% to 5.10%). Additionally, the weight loss of titanium chamber approached 20%, which is considered as an abort criterium.

Similar beneficial results for the PEEK chamber were found for faeces corticosterone metabolites (FCMs; Fig. 4B). In the PEEK group, only two animals experienced a slight increase in FCMs during the early phase (147%/121–281% p = 0.13). In contrast, early phase FCMs in mice with titanium chambers increased fourfold compared to baseline (baseline: 100%, early phase: 368%/248–587%; p < 0.05). In the middle and late phase, FCM values in both groups returned to the baseline (Fig. 4B). The direct comparison between PEEK and titanium chambers revealed a significant increase of titanium chamber FCMs in the early phase (PEEK: 147%/121–281%; titanium: 368%/248–587%, p < 0.05), while FCMs were comparable in the middle (PEEK: 130%/37–220%; titanium: 101%/59–203%, p = 0.31) and late phase (PEEK: 108%/81–395%; titanium: 90%/54–196%, p = 0.85).

**Figure 5:**
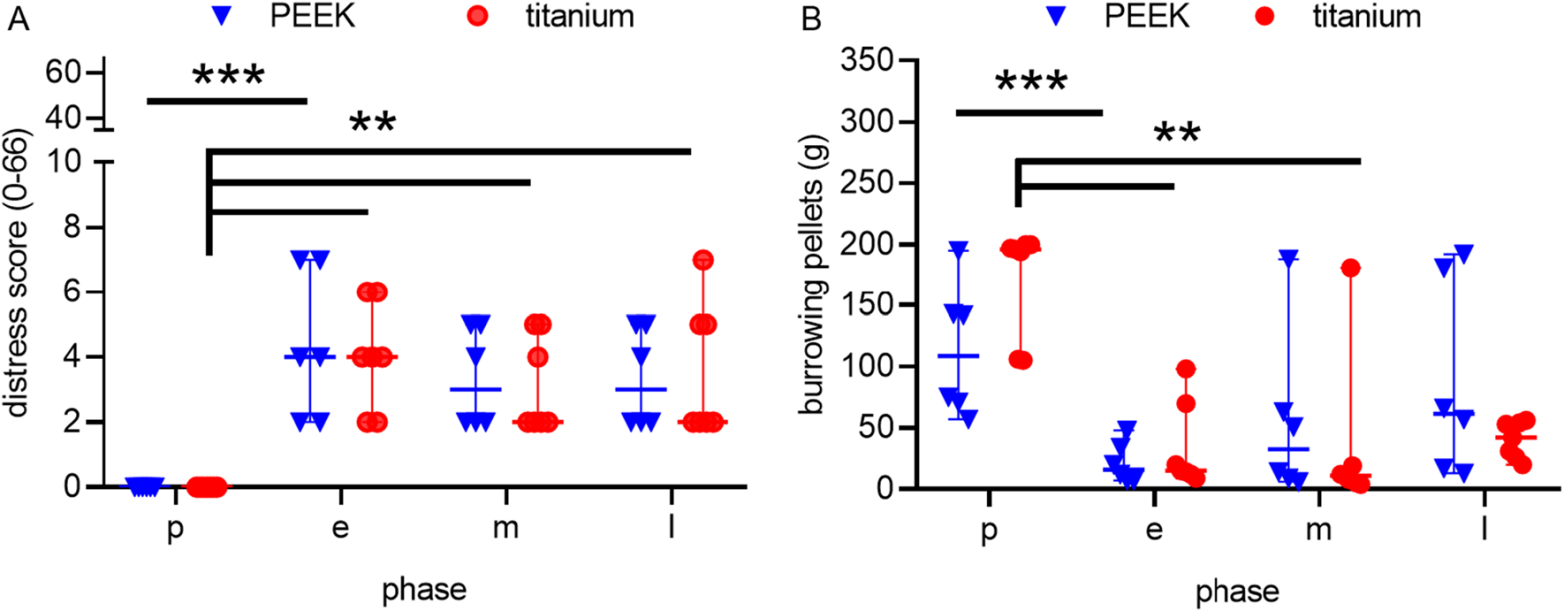
Distress Score and burrowing activity after dorsal skinfold chamber implantation. Individual values are given over time in a preoperative phase (p), early postoperative phase (e), intermediate postoperative phase (m) and late postoperative (l) phase. Specific distress scoring and burrowing assessment did not show differences between the titanium and the PEEK chamber. Distress score (**A**) and burrowing activity (**B**) at each time point were compared by Friedman test followed by Dunn’s correction and the difference between each group was analysed by unpaired t test (Holm-Sidak). **p < 0.05, ***p < 0.05; PEEK: n = 6, titanium: n = 6.

Specific distress scoring and burrowing assessment did not show differences between titanium and the PEEK chambers (Fig. 5). Distress values remained low, with maximum 7/66 points for PEEK and 6/66 points for titanium chambers (p > 0.8268). In both groups, the distress score increased slightly in the early phase and declined in the middle and late phase. Likewise, burrowing started with relatively high baseline values (PEEK: 108 g/57–195 g; titanium: 196 g/105–200 g, p = 0.06). Postoperative burrowing decreased significantly in both groups (PEEK: p < 0.05; titanium: p = 0.05, vs. baseline). Burrowing remained decreased markedly throughout the observation time in both groups (PEEK: 61 g/13–192 g; titanium: 36 g/20–56 g).

In summary, the PEEK chamber is easily available and maintains the high quality of intravital vascular imaging. The PEEK chamber's flat and lightweight design can reduce animal distress and prolong the maximum duration of the experiment.

## Discussion

The dorsal skinfold chamber is a major model for repetitive examination of vascular changes and inflammation. However, the dorsal skinfold chamber causes considerable stress for the test animals. Therefore, the model is not only criticized by animal welfare groups. This criticism is understandable, since many improved models have already been published, but mainly the classical titanium chamber is used^17,23,24,27–29^. For the widespread establishment of plastic chambers, a simple manufacturing protocol has been lacking on the one hand and proof of superiority in distress reduction on the other.

Here, we present a simple 3D-printed model made of PEEK. We show that the PEEK chamber reduces distress and extends maximum observation time.

The surgical demands and the quality of intravital microscopy are equivalent in PEEK and titanium chambers. When implanting the PEEK chamber, penetration of the skinfold with screws can be omitted, because of the chamber's light weight. In titanium chambers, screws cause penetrating holes at the base of the skinfold, which are associated with chamber tilting. The PEEK chamber is fixed by tear resistant sutures, which the test animal cannot reopen (FibreWire, Arthrex, Munich, Germany). These sutures cause less trauma than previously used screws. After chamber implantation, the experiments run without differences to titanium chambers. In particular, there are no differences in the quality of the repetitive intravital fluorescence microscopy.

The most common complication of dorsal skinfold chamber experiments is lateral tilting of the chamber during the second week^17^. By the third week, 50% of the titanium chambers tilt to a position of > 90°, which causes animal immobilization. In all PEEK chambers, tilting remained below 90° for three weeks. The only PEEK chamber that had a tilting of 50° in the second week remained constant in the following week. This is clearly because of the reduced weight. In contrast, titanium chambers continued tilting over time. Therefore, the stable position of PEEK chambers could extend the maximum duration of future experiments to four or five weeks.

Besides decreased tilting in the third week, the use of PEEK chambers also reduced distress for the experimental animals in the postoperative period. Postoperative weight loss is significantly higher in titanium chamber animals and does not return to baseline values over three weeks. With PEEK chambers, on the other hand, the test animals reach their original weight as early as the second week. Consistent with our data, previous research described up to 15% postoperative weight loss for titanium chambers and decreased weight loss for non-metal dorsal skinfold chambers^30–32^.

This difference in stress was confirmed by FCM measurement, a non-invasive measure of adrenocortical activity^33^. FCMs increased significantly after implantation of a titanium chamber while only a slight increase in FCMs was observed for PEEK chambers. Therefore, postoperative stress was primarily related to the titanium chamber and not to the surgery itself. In the intermediate and late phase, FCMs returned to baseline values in both groups. The design of future titanium chamber experiments should consider increased postoperative stress as a potential bias^34^.

In contrast to FCMs and body weight changes, mice in both groups did not differ in burrowing activity nor distress score. The distress score remained at a low level after the operation. The postoperative increase of 7/66 points in the distress score was statistically significant. However, the values remained in the lower range of the score.

To our knowledge, this study is the first to investigate the distress of laboratory animals with dorsal skinfold chambers. Despite the lack of data on animal distress, many chamber improvements have already been published. These improvements were supposed to reduce animal distress and to enable MRI imaging. A simple development is a smaller titanium frame with an equally large observation window^29^. These smaller titanium chambers are sold commercially in the United States (small dorsal kit SM100, APJ Trading Co., Ventura, CA, USA). Schreiter et al*.* describe the advantages of a self-designed small titanium chamber: postoperatively no recovery period was necessary, younger animals could be used and their stress was supposed to be reduced^1^. However, titanium chambers are not MRI compatible and screw fixation is necessary. Furthermore, titanium is difficult to process and cannot be manufactured in life science facilities.

Innovative developments are chambers made of plastic, which have been used for years in Japan and the US. The first plastic chamber made of Duracon was described in 2003 by Ushiyama et al*.*^17^. The publication illustrated reduced tilting and supposed distress reduction, because of the lightweight Duracon material. However, quantification of tilting and distress was not performed. In addition, these early plastic chambers continued to use screw fixation^17,35^. These screws penetrate the skin and cause large wounds at the chamber basis.

A further weight reduction was achieved by using thermoplastic PEEK. PEEK is characterized by a high flexural modulus (3738 MPa) and tensile strength (100 MPa) compared to Duracon (2500 MPa, 87 MPa) and acrylic glass (3210 MPa, 75 MPa)^36^. PEEK can therefore resist the bite of rodents. Furthermore, PEEK can be fabricated with additive manufacturing processes, which results in further advantages such as a high degree of geometric freedom, low production costs and the flexibility regarding the unique or single-part production^37^.

The first lightweight PEEK chambers were introduced by Gaustad et al*.* and Seynhaeve et al*.*^23,28^. The PEEK chamber weight was as low as 1 g and 1.1 g, respectively. The chambers were fixed using sutures (Gaustad) or small screws (Seyhaeve). Mice fitted with the chambers showed a full capacity of motion, climbed, and gained weight as mice without chambers. We observed similar positive effects for PEEK chambers (body weight, climbing, mobility). In addition, we verified reduced distress using a standardized protocol. We have repetitively measured chamber tilting and found that the PEEK chambers significantly reduce tilting in the third week of the experiment. Lightweight chambers with reduced lateral tilting enable increased observation times of up to one month^27^. This is especially relevant for biomaterial and oncology research: dorsal skinfold chamber observation times of three to five weeks could enable to study long-term biomaterial integration (fibrosis, giant cell formation, implant vascularization)^38^. In oncology, longer observation times could significantly improve the model, since the growth of tumor cells already preoccupies large parts of the current maximum observation time^39^.

The reduction in tilting was significant, although the measurement method had limitations, as the chamber position depends on the body position. The measurement was performed on anaesthetized animals in an upright position with all feet on the ground. However, when positioning the animals, slight deviations of the angles occurred.

Another limitation of the study is that the standardized distress score does not focus on the immobilization of the test animals. However, the impediment of free movement, because of tilting and chamber weight, probably represents the main restriction for the experimental animals. Electronically recording of the animal mobility through tracking systems or recording of the time spent climbing the cage could increase the power of the stress analysis. However, using standardized distress scores enables comparisons to previous experiments.

The low number (n = 6) of test animals may be considered another limitation of the study. However, the PEEK chamber was significantly superior in major outcomes, such as tilting and weight loss. Therefore, no additional experimental animals had to be included. Another limitation related to the study design is that the PEEK and the titanium chambers have different sizes. Hence, all conclusions are related to the design (height, weight), but not to the material (PEEK vs. titanium). Low and lightweight titanium chambers may also decrease animal distress compared to large standard titanium chambers. Yet, we consider PEEK a more suitable material because it increases availability, enables imaging, and decreases costs. These main advantages render a light titanium group obsolete.

### Conclusion

In experiments with dorsal skinfold chambers, the animals are particularly stressed by classical titanium chambers. This setup should be revised, in the context of 3R. Despite the development of smaller and lighter chambers, most dorsal skinfold chamber experiments in recent years have continued to use titanium chambers. We have shown that lighter chambers can significantly reduce animal distress and even extend the maximum experiment duration. Chambers made of PEEK are particularly suitable for this purpose: They are autoclavable, sufficiently stable to withstand rodents, inexpensive, and widely available through 3D printing.

## Methods

### PEEK chamber printing

The PEEK chamber was designed for geometric shape improvement, weight reduction, and optimization for additive manufacturing using SolidWorks (Dassault Systèmes, Waltham, MA, USA). The Fused Filament Fabrication (FFF) process and the printer Minifactory ultra (miniFactory Oy LTD, Seinäjoki, Finland) were used for chamber printing. The design (*.stl file) was imported to Simplify3d (Simplify3d, Ohio, US). Biocompatible and steam sterilizable PEEK filament Intamsys Funmat HT (INTAMSYS Technology Co. Ltd, Shanghai, China) with a flexural modulus of 3738 MPa and tensile strength of 100 MPa was chosen. The material-dependent printing parameters were 230 °C chamber temperature, 190 °C bed temperature, 420 °C nozzle temperature 0.4 mm nozzle diameter, and 18 mm/s printing speed. Slicing was done according to the manufacture settings with a corresponding layer thickness of 250 µm. A brim of 3 mm gave optimal hold to the chamber on the printer bed. To ensure a plane chamber surface for skin contact side, the bottom of the chamber was placed on the printer glass bed for slicing. An extrusion multiplexer of 1.02 was set to fill production-related gaps between the filaments in the x–y direction (see supplementary PEEK chamber 3D printing protocol). Irregularities on the top side (window side) were ground manually after the printing process (printed chamber before polishing shown in supplementary Figure S1 and polished surface shown in Fig. 2B). Seven holes of 1 mm diameter were drilled into the frames for suture chamber fixation using a template. Before experimental use, the chamber was visually proved and post processed by steam sterilization.

### Animals and ethics statement

All in vivo experiments were conducted in accordance with the German legislation on protection of animals (7221.3-1-012/20) and the NIH Guide for the Care and Use of Laboratory Animals (Institute of Laboratory Animal Resources, National Research Council). Male hairless SKH1/hr mice (6–10 weeks of age and weight of 25–30 g) were used for all experiments. The animals were housed individually in a specific pathogen-free facility with a twelve-hour light–dark cycle and access to standard laboratory chow and water ad libitum.

### Study design

**Figure 6:**
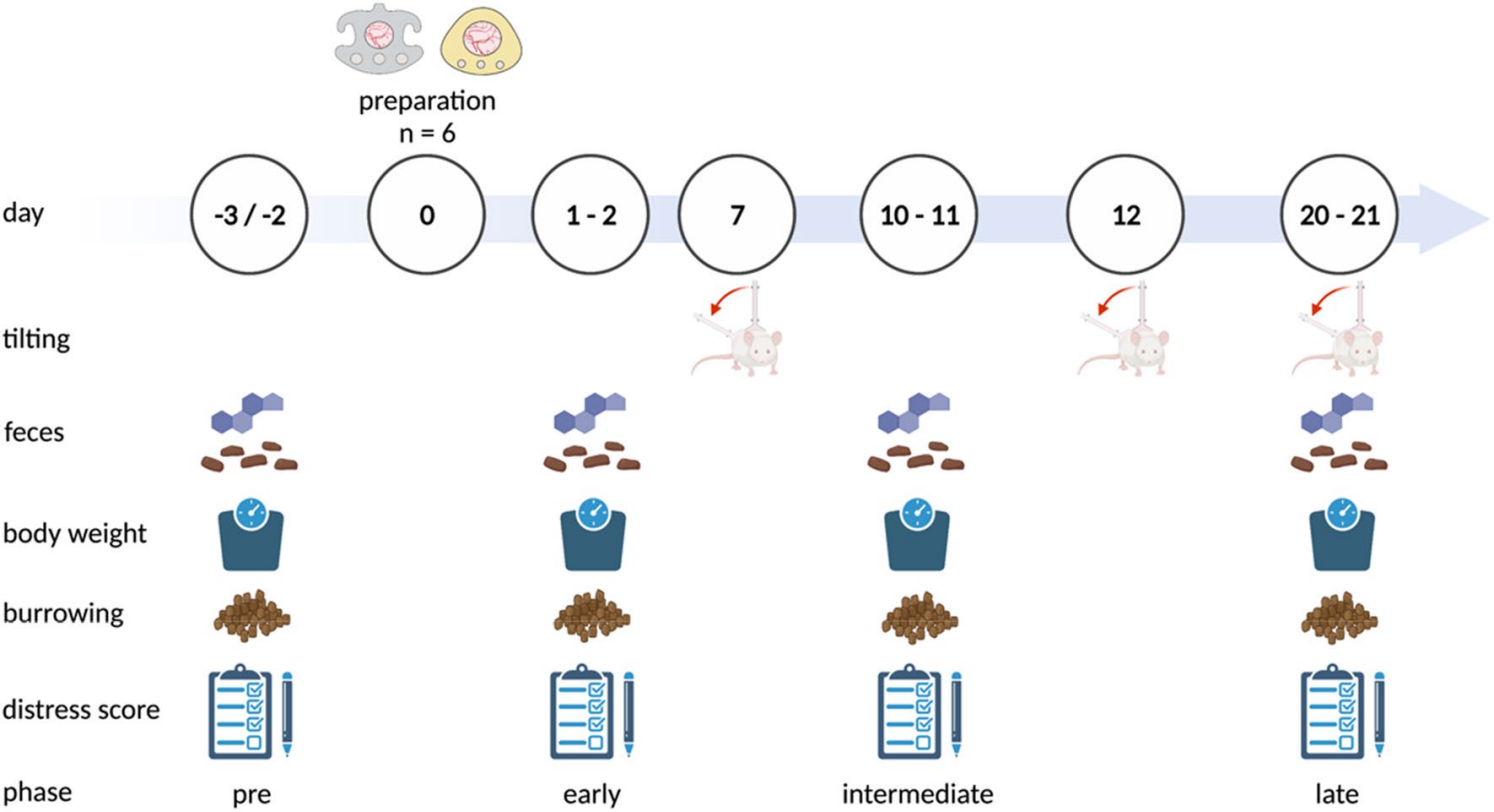
Experimental design. The dorsal skinfold chambers were implanted on day 0. Tilting angles were assessed on day 7, 12 and 21. Collection of faeces, body weight measurement, burrowing analysis and distress scoring were performed in a preoperative, early, intermediate, and late postoperative phase. Figure created with BioRender.com.

Twelve mice were randomly allocated to two experimental groups: titanium chamber and PEEK chamber. Each animal was one experimental unit and examined independently using body weight, faecal corticosterone metabolites (FCMs), burrowing activity and clinical distress scores on days 1/2, 10/11 and 20/21 after dorsal skinfold chamber preparation. Following distress and tilting measurements on day 21, mice were sacrificed (Fig. 6).

### Experimental procedures

Dorsal skinfold chamber implantation: Mice were anesthetized by an intraperitoneal (ip) injection of ketamine/xylazine (90/10 mg/kg bw) and positioned on a heating pad (37.8 °C). Microsurgery for dorsal skinfold titanium chamber implantation has been described before^5^. The significant change in the proposed model is the preparation without screws: After disinfection of the dorsal skin (Octeniderm, Schülke & Mayr GmbH, Norderstedt, Germany) and marking of the median line, a skin bilayer was stretched in the median line. Subsequently, the back of the PEEK chamber was sutured to the skin fold through the preformed holes (FiberWire, Arthrex, Munich, Germany). The preparation area was color-marked and the microsurgical preparation of the front side was performed. After completion of the preparation, the front of the chamber was placed congruently and fixed by three sutures connecting both chamber frames (Fig. 2).

### Chamber tilting

For chamber tilting analysis, animals were sedated in an isoflurane chamber for approximately ten seconds on days 7, 12, and 20/21. The sedated animals were positioned upright, with all feet on the floor. In the upright position, the animals were photographed from behind. In Photoshop software (Adobe Inc., San José, U.S.), the lateral tilting angle was measured in degrees of deviation from a vertical line.

### Body weight

The body weight was measured on a scale (EMB 200-2, Kern & Sohn, Balingen, Germany) at 9:00–9:30 am. Percent change in body weight was determined by comparison with body weight in the preoperative phase.

### Distress score

Since handling may affect animal distress, the distress score was assessed before weighting. The distress score sheet comprises body weight, general condition, spontaneous behaviour, flight behaviour and process-specific criteria, as previously published^40^. (Suppl. Tables S2 and S3).

### Burrowing

To quantify the burrowing activity, a tube (length: 15 cm, diameter: 6.5 cm) filled with 200 ± 1 g of food pellets (ssniff Spezialdiaeten GmbH, Soest, Germany) was placed in the left back corner of the cages 3 h before the dark phase at 04:00–04:10 pm. Despite the implanted chambers, mice had free access to these pellets throughout the whole observation time. The weight of the food pellets (g) left in the tube was measured on the next day.

### Faecal corticosterone metabolites (FCMs)

After weighting, the bedding with old faeces was removed and replaced by fresh beddings. After 24 h, at least 400 mg faeces were collected per cage. The faeces were dried for 4 h at 65 °C and kept at − 20 °C until further processing. 50 mg of homogenized, dried faeces were extracted with 1 mL of 80% methanol and FCMs analyzed with a 5α-pregnane-3β, 11β, 21-triol-20-one enzyme immunoassay^41,42^. FCMs were evaluated blinded, and the percentage of FCMs was determined by comparison to respective FCM concentrations in the pre-operative phase.

### Intravital microscopy

Representative intravital microscopy was performed on day 3. Mice were anesthetized and placed on a plexiglass pad with integrated heating. For the visualization of the microvascular system fluorescein isothiocyanate–labeled (FITC)-dextran (0.05 ml, 5%, MW: 150 kD) was injected into the lateral tail vein (or into the retrobulbar venous plexus if tail vein injection failed). Intravital microscopy was performed with 50-, 100- and 200-fold magnification using an Axiotech vario microscope (Carl Zeiss AG, Oberkochen, Germany) with a 100-W HBO mercury lamp with a blue filter (excitation, 450–490 nm; emission, 520 nm) The microscopic images were recorded on DVD (DMR-EX99V, Panasonic, Kadoma, Japan) using a charge-coupled video camera (FK 6990A-IQ, Pieper, Berlin, Germany) for off-line evaluation.

### Statistics

Data were graphed and analyzed with GraphPad Prism (version 8.0.1, GraphPad Software Inc., San Diego, CA, U.S.) and were presented as median and 95% confidence interval. The characteristics of data were assessed by Shapiro Wilk test. When analyzing the influence of time on the dependent variables, a Friedman Test was performed (corrections of multiple comparisons using Dunn test) in tilting angles, burrowing activity, percentage of FCMs and distress score, and a one-way repeated measure ANOVA was performed (corrections of multiple comparisons using Tukey test) in the percentage of body weight change analysis. When analyzing the influence of the chambers on the dependent variables, a Mann Whitney Rank sum test (for non-parametric data) or unpaired t test (for parametric data) was used. Differences with p < 0.05 were considered significant. Data are given as median/95% confidence interval.

## Supplementary Information

Supplementary Information.

Supplementary Tables.

Supplementary Figure S1.

## Data Availability

The datasets generated and analyzed during the current study are available from the corresponding author on reasonable request.
