# Supplementary material for: 3D-printed lightweight dorsal skin fold chambers from PEEK reduce chamber-related animal distress
Source: Sci Rep. 2022 Jul 8;12:11599. doi: 10.1038/s41598-022-13924-5 (PMC9270450; doi:10.1038/s41598-022-13924-5)
Supplement: Supplementary file 1 — Supplementary Information. [file 41598_2022_13924_MOESM1_ESM.docx]

**PEEK chamber 3D printing protocol**

M106 P2 S1

M106 P3 S0.6 F5

M572 D0:1 S0.06 ;Pressure advance for PEEK CF

; Set extruder temperatures

G10 P0 S[extruder0_temperature] R[extruder0_temperature]

G10 P1 S[extruder1_temperature] R[extruder1_temperature]

; Set Chamber temperature

M141 S[extruder5_temperature]

T99 ; Disable all tools

T0 ; Choose the right extruder

G92 E0 ; zero extruder

G1 Z5 F5000 ; Raise bed into position

; M42 P5 S1 ; Enable Vacuum (disabled for now)

; END OF STARTING SCRIPT

; ENDING SCRIPT

G92 E0 ; Set active tool's extruder position to 0

M83 ; Set extruder to relative mode

G91 ; Set axis to relative mode

G1 E-3 F600 ; Retract 3mm

T99 ; Disable tools

G28 Z0 ; Home Z

G90 ; Absolute mode

G10 P0 S0 R0 ; Turn Extruder OFF

G10 P1 S0 R0 ; Turn Extruder OFF

G28 X0

G1 Y180 F4800 ; Part to Front

M42 P4 S0 ;Turn off VACUUM

M42 P5 S1 ;Open VACUUM release valve

G4 S1 ;Wait 1s

M42 P5 S0 ;Close VACUUM release valve

M106 P0 S0 ; Turn off hot blower

M140 S0 ; Turn off bed

M141 S0 ; Turn off chamber
