# Supplementary material for: 3D-printed lightweight dorsal skin fold chambers from PEEK reduce chamber-related animal distress
Source: Sci Rep. 2022 Jul 8;12:11599. doi: 10.1038/s41598-022-13924-5 (PMC9270450; doi:10.1038/s41598-022-13924-5)
Supplement: Supplementary file 2 — Supplementary Tables. [file 41598_2022_13924_MOESM2_ESM.docx]

| **year** | **chamber material** | **research field** | **authors** | **DOI** |
| --- | --- | --- | --- | --- |
| 2021 | titanium | cancer | Senger et al. | 10.1016/j.aanat.2021.151746 |
| 2021 | titanium | tissue engineering | Nalbach et al. | 10.1177/20417314211035593 |
| 2021 | titanium | endometriosis | Nenicu et al. | 10.1093/humrep/deab139 |
| 2021 | titanium | microcirculation | Eckrich et al. | 10.1016/j.redox.2021.102063 |
| 2021 | titanium | endometriosis | Rudzitis-Auth et al. | 10.1111/bph.15601 |
| 2021 | polyoxymethylene | drug release | Nawijn et al. | 10.3791/62251 |
| 2021 | titanium | tissue engineering | Schreiter et al. | 10.1016/j.mvr.2021.104131 |
| 2021 | titanium | tissue implantation | Schlachtenberger et al. | 10.1093/icvts/ivaa308 |
| 2021 | titanium | tissue engineering | Hussain et al. | 10.1007/s13770-020-00325-w |
| 2021 | titanium | tissue engineering | Später et al. | 10.1177/20417314211000304 |
| 2021 | titanium | cancer | Perrone et al. | 10.1016/j.jconrel.2020.11.020 |
| 2021 | titanium | microcirculation | Kihm et al. | 10.1016/j.bpj.2020.12.012 |
| 2021 | titanium | microcirculation | Kang et al. | 10.4266/acc.2020.00969 |
| 2021 | titanium | inflammation | Sok et al. | 10.1016/j.biomaterials.2020.120475 |
| 2021 | titanium | tissue engineering | Wagner et al. | 10.1038/s41440-020-0524-z |
| 2020 | titanium | wound infection | Schreiter et al. | 10.3205/iprs000150 |
| 2020 | titanium | tissue implantation | Dau et al. | 10.1002/jbm.a.36989 |
| 2020 | PEEK | cancer | Tong et al. | 10.1177/1758835920965853 |
| 2020 | titanium | tissue engineering | Später et al. | 10.1021/acsbiomaterials.0c00741 |
| 2020 | titanium | tissue engineering | Dudenhöffer et al. | 10.1055/s-0038-1675595 |
| 2020 | titanium | tissue implantation | Menger et al. | 10.1007/s00592-020-01512-w |
| 2020 | titanium | endometriosis | Rudzitis-Auth et al. | 10.1111/bph.15044 |
| 2020 | titanium | endometriosis | Zhao et al. | 10.1016/j.fertnstert.2019.12.035 |
| 2020 | titanium | tissue engineering | Später et al. | 10.1016/j.actbio.2020.03.018 |
| 2020 | titanium | tissue engineering | Später et al. | 10.1177/2041731420911816 |
| 2020 | titanium | skin graft | McLuckie et al. | 10.1016/j.actbio.2020.01.050 |
| 2020 | titanium | tissue implantation | Menger et al. | 10.1111/bph.14925 |
| 2020 | titanium | thrombosis | Ampofo et al. | 10.3390/md18020111 |
| 2020 | titanium | wound healing | Gniesmer et al. | 10.1371/journal.pone.0227563 |
| 2020 | titanium | microvasculation | Kelly et al. | 10.1002/lsm.23186 |
| 2020 | titanium | tissue engineering | Jehn et al. | 10.1016/j.mvr.2019.103925 |
| 2019 | titanium | cancer | Ludwig et al. | 10.1016/j.canlet.2019.10.010 |
| 2019 | titanium | microvasculation | Lima et al. | 10.1016/j.mvr.2019.103909 |
| 2019 | titanium | tissue implantation | Nalbach et al. | 10.1007/s00441-019-03048-0 |
| 2019 | titanium | tissue engineering | Laschke et al. | 10.22203/eCM.v038a13 |
| 2019 | titanium | tissue engineering | Laschke et al. | 10.1177/2041731419879837 |
| 2019 | titanium | topical formulations | Dahmke et al. | 10.1111/exd.13983 |
| 2019 | titanium | anti-angiogenesis | Rieck et al. | 10.1016/j.jconrel.2019.05.031 |
| 2019 | titanium | tissue engineering | Laschke et al. | 10.2217/rme-2018-0164 |
| 2019 | titanium | inflammation | Ampofo et al. | 10.1038/s41598-019-42465-7 |
| 2019 | titanium | tissue engineering | Cottler et al. | 10.1097/PRS.0000000000005410 |
| 2019 | titanium | tissue implantation | Regelin et al. | 10.1038/s41598-019-41590-7 |
| 2019 | titanium | tissue engineering | Laschke et al. | 10.1002/term.2774 |
| 2018 | titanium | tissue engineering | Hessenauer et al. | 10.1016/j.actbio.2018.10.004 |
| 2018 | titanium | tissue engineering | Später et al. | 10.1186/s13036-018-0118-3 |
| 2018 | titanium | cancer | Uhl et al. | 10.1007/s10456-018-9633-6 |
| 2018 | titanium | cancer | Mussawy et al. | 10.1186/s12885-018-4905-5 |
| 2018 | titanium | skin graft | Lacerda et al. | 10.3389/fphar.2018.01015 |
| 2018 | titanium | wound healing | Sorg et al. | 10.1016/j.ultrasmedbio.2018.03.006 |
| 2018 | titanium | tissue engineering | Karschnia et al. | 10.22203/eCM.v035a19 |
| 2018 | steel wire | sickle cell disease | Gu et al. | 10.1016/j.exphem.2018.01.002 |
| 2018 | titanium | tissue engineering | Laschke et al. | 10.1002/term.2591 |
| 2018 | titanium | endometriosis | Körbel et al. | 10.1007/s10456-017-9580-7 |
| 2018 | PEEK | cancer | Seynhaeve et al. | 10.3791/55115 |
| 2018 | titanium | tissue engineering | Später et al. | 10.1111/wrr.12621 |
| 2018 | titanium | tissue engineering | Später et al. | 10.1002/jbm.b.33813 |
| 2017 | titanium | red blood cells | Danielczok et al. | 10.3389/fphys.2017.00979 |
| 2017 | titanium | wound healing | Xing et al. | 10.1117/1.JBO.22.11.115005 |
| 2017 | titanium | inflammation | Molás et al. | 10.1016/j.toxicon.2017.06.009 |
| 2017 | titanium | wound healing | Klein et al. | 10.1088/1748-605X/aa7e80 |
| 2017 | titanium | microcirculation | Pi et al. | 10.1186/s12886-017-0556-6 |
| 2017 | titanium | tissue engineering | McLuckie et al. | 10.1002/jbm.a.36108 |
| 2017 | titanium | tissue engineering | Körbel et al. | 10.22203/eCM.v034a04 |
| 2017 | titanium | microcirculation | Mussawy et al. | 10.1371/journal.pone.0183186 |
| 2017 | titanium | model refinement | Schreiter et al. | 10.3205/iprs000112 |
| 2017 | titanium | inflammation | Ampofo et al. | 10.1016/j.jss.2017.03.019 |
| 2017 | titanium | cancer | Zimmerer et al. | 10.1016/j.mvr.2017.03.006 |
| 2017 | titanium | thrombosis | Grambow et al. | 10.1080/09537104.2016.1235693 |
| 2017 | titanium | cancer | Senger et al. | 10.1007/s10585-017-9852-z |
| 2017 | NA | sickle cell disease | Belcher et al. | 10.1089/ars.2015.6571 |
| 2017 | titanium | cancer | Stéphanou et al. | 10.1016/j.jtbi.2017.02.018 |
| 2017 | titanium | tissue engineering | Später et al. | 10.22203/eCM.v033a20 |
| 2017 | titanium | tissue engineering | DeGeorge et al. | 10.1097/PRS.0000000000002992 |
| 2017 | titanium | cancer | Melsens et al. | 10.1159/000452741 |
| 2017 | NA | cancer | Maeda et al. | 10.1016/j.ijrobp.2016.09.005 |
| 2017 | titanium | tissue engineering | Frueh et al. | 10.1016/j.jid.2016.08.010 |

**Supplementary table S1.** Review over chamber application between 2017 and 2021.

|  |  |
| --- | --- |
| Observation | **Score** |
| I Body weight  I-a decreased > 10% (compared to initial weight)  I-b decreased > 20% (compared to initial weight) | 2  5 |
|  |  |
| II General condition  II-a tooth displacement, too long teeth  II-b fur dull, ruffled or untended  II-c eyes unclear or squinted  II-d untended orifices of the body  II-e abnormal posture  II-f dehydration  II-g short spasms or temporary paralysis symptoms  II-h persistent (>30’) cramping or paralysis  II-i abnormal respiratory sounds or animal feels cold | 1 (A)  2  2  3  3  3  3  5  5 |
|  |  |
| III Spontaneous behavior  III-a the animal is passive or overactive  III -b pronounced apathy, hyperkinetic, or isolation  III -c squeaking due to pain  III -d self-mutilation | 2  4  5  5 |
|  |  |
| IV Flight behavior after contact  IV-a animal is passive or overactive  IV-b distinct apathy or hyperkinetic | 2  5 |
|  |  |
| V Process-specific criteria  V-a wound healing disorder  V-b opening of the sutures by biting  V-c local inflammation  V-d ascites | 2  1 (B)  2  4 |
|  |  |
| Total score | 0-66 |
| Supplementary Table S2. Distress score on mice. Score points are stated per line as soon as one criteria applies. Even with several positive results per line, there is no addition of the points per line. | |

| Single score | Total score | Distress level | Measures |
| --- | --- | --- | --- |
| A |  | mild | Anesthetize animal and shorten teeth. Document it. |
| B |  | mild | Inform the person in charge of the experiment. If necessary, anesthetize the animal and close the wound. Document it. |
| 1 |  | mild | Inform the person in charge of the experiment. A sufficient frequency of observation is necessary, consider treatment options and document it. |
| 2-4 |  | moderate | Inform the person in charge of the experiment. Daily observation of the animal is necessary, consider treatment options and document it. |
| 5 |  | severe | In agreement with the person in charge euthanasia (preferably painless after anesthesia) has to be performed. Document it. |
|  | 3-4 | mild | Inform the person in charge of the experiment. Daily observation of the animal is necessary, consider treatment options and document it. |
|  | 5-15 | moderate | Inform the person in charge of the experiment. Euthanasia or treating the animal plus daily observation of the animal is necessary. Document it. |
|  | >15 | severe | In agreement with the person in charge euthanasia (preferably painless after anesthesia) has to be performed. Document it. |

**Supplementary Table S3.** Consequences according to distress score.
