# Supplementary material for: 3D-printed lightweight dorsal skin fold chambers from PEEK reduce chamber-related animal distress
Source: Sci Rep. 2022 Jul 8;12:11599. doi: 10.1038/s41598-022-13924-5 (PMC9270450; doi:10.1038/s41598-022-13924-5)
Supplement: Supplementary file 3 — Supplementary Figure S1. [file 41598_2022_13924_MOESM3_ESM.docx]

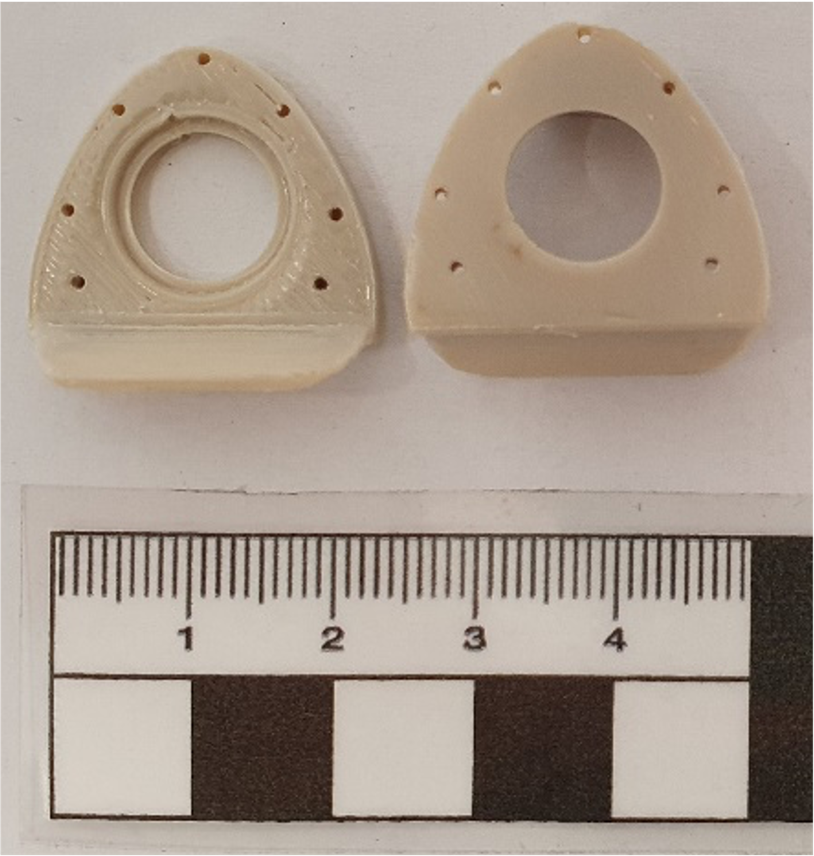


**Supplementary Figure 1**. Raw PEEK chamber following extrusion printing. To correct for irregularities the surface was ground before experimental use.
